# The association between animal flesh foods consumption and semen parameters among infertile Iranian men: a cross-sectional study

**DOI:** 10.1186/s12937-020-00633-w

**Published:** 2020-10-06

**Authors:** Reza Ghiasvand, Laleh Dehghan Marvast, Seyyed Payam Shariatpanahi, Makan Pourmasoumi, Cain C. T. Clark, Farahnaz Haeri

**Affiliations:** 1 Department of Community Nutrition, School of Nutrition and Food Science, Isfahan University of Medical Sciences, Isfahan, Iran; 2 Andrology Research Center, Yazd Reproductive Sciences Institute, Shahid Sadoughi University of Medical Sciences, Yazd, Iran; 3 Department of Epidemiology and Biostatistics, School of Health, Isfahan University of Medical Sciences, Isfahan, Iran; 4 Gastrointestinal and Liver Diseases Research Center, Guilan University of Medical Sciences, Rasht, Iran; 5 Centre for Intelligent Healthcare, Coventry University, Coventry, CV1 5FB UK

**Keywords:** Diet, Male infertility, Semen analysis, Meat intake

## Abstract

**Background:**

Previous studies have demonstrated the association between dietary patterns and semen quality indicators, but research on the possible association between animal flesh foods consumption and semen quality is limited. Therefore, this study was conducted to investigate the association between animal flesh foods consumption with semen quality.

**Methods:**

In this cross-sectional study, 400 newly-identified (< 6 months) infertile men, as diagnosed by an andrologist, were recruited into the study. Dietary intake was assessed by using a semiquantitative Food Frequency Questionnaire. The total meat consumption was defined as the sum of red meat, poultry, fresh fish, canned fish, processed meats, and organ meats in the diet. A linear mixed model was used to assess the relationship between meat consumption and semen quality indicators of participants.

**Results:**

Consumption of canned fish was inversely related to sperm immotility. Compared with the men in the lowest quartile of canned fish intake, those in the highest quartile had a lower sperm immotility [lowest quartile: 52.5%; (95% CI: 47–57) vs 47.4%; (95% CI: 43–51) P-trend = 0.026]. Similarly, a trend toward an inverse significant association between fresh fish intake and sperm immotility was observed (P-trend = 0.074). In contrast, fresh and canned fish intake was unrelated to other outcomes of sperm quality (P-trend > 0.05). No association was found between consumption of processed red meat, red meat, poultry, and organ meat, and semen quality indicators (P-trend > 0.05).

**Conclusions:**

We found that consumption of canned fish is associated with a lower percentage of immotile sperm, whilst a high consumption of fresh fish increased the percentage of immotile sperm in Iranian infertile men. Further studies are recommended in this regard.

## Background

Infertility affects 7% of the total male population, globally [1], and more than 25% of infertility is caused by a decrease in semen quality [2]. According to a meta-analysis, involving 185 studies and 42,000 men, semen quality has decreased over the last 40 years [3]. Although according to previous studies male infertility might be due to anatomical disorders such as varicocele, obstruction of the ducts, or ejaculatory disorders [4, 5], about 40 to 90% of the causes of male infertility are attributed to a decrease in semen quality and abnormal sperm health indicators [4, 5]. Indeed, several reasons have been suggested for semen quality declination, but smoking, alcohol consumption, pesticides in food, unhealthy eating habits, and inadequate intake of many essential micronutrients and vitamins are regarded as the main causes of this reduction [6]. Indeed, existing studies have demonstrated a link between infertility and lifestyle patterns, inclusive of dietary habits [7, 8]. Rapid changes in dietary behavior, such as the increased prevalence of unhealthy dietary patterns, characterized by lower consumption of antioxidant-rich foods, such as fruits and vegetables and higher intake of trans fatty acids, saturated fat, and sodium, have impacted reproductive health [7]. It has been shown that high consumption of poultry, skimmed milk, and seafood are associated with a significantly lower risk of asthenozoospermia [7]. Furthermore, there is evidence to suggest that not only could food intake and human nutrition be associated with poor semen quality, but also could affect the quality of semen in men undergoing IVF/ICSI procedures [9–11]. Despite promising result from the aforementioned studies, result in this area is not conclusive. While some studies have shown an association between flesh animal food consumption and variable related to infertility such as semen quality, others failed to find any association in this field [12–14]. As a consequence, many researchers are still assessing the hypothesis which indicates that animal flesh foods consumption is associate with male infertility. This discrepancy between finings needs further studies to make a conclusive evidence-based decision. In addition, data in this area is a few among the Iranian population, as genetics is an undeniable factor for infertility; so that, there is necessary to more investigation in this area on Iranian men with this malignancy. Therefore, in the present study, we sought to examine the relationship between animal flesh foods consumption and sperm quality indicators in infertile men.

## Materials and methods

### Study population

In this cross-sectional study, 400, newly diagnosed (< 6 months), infertile men, according to clinical diagnosis by an andrologist, participated in this study, between July 2019 and December 2019, from Yazd Reproduction Research Institute. Inclusion criteria included: individuals with age between 20 to 55 years, and abnormal semen parameters, including sperm count less than 15 million per milliliter and/or normal morphology less than 4% and/or semen volume less than 1.5 ml and/or progressive motility less than 40% [15]. Also, exclusion criteria included: chronic diseases, testicular atrophy, ejaculatory disorder, hypospadiasis, stenosis, varicocele, adherence to specific diets, such as weight loss diet, diet for athletes/athletic competition, or any other diets which change the usual dietary intake of individuals, non-response to more than 35 items of food frequency questionnaire, and under-reporting and over-reporting of energy intake (less than 800 and more than 4200) (Supplemental Figure 1). General and dietary information was collected by a trained nutritionist. All participants signed informed consent preceding study commencement.

### Physical examination and lifestyle variable

Physical activity data were collected using a validated and reliable questionnaire (International Physical Activity Questionnaire) [16]. The IPAQ provides information about levels of inactivity, moderate activity, strenuous activity, and walking. In addition, we gathered the data regarding frequency (days per week) and duration (minutes per day) for all types of activities.

Socioeconomic status (SES) of the study participants was determined according to variables, such as homeownership (landlord-tenant), washing machine and dishwasher (yes-no), number of overseas trips, has a car (yes-no), individual occupation, and education (number of years of study).

### Anthropometric data

Anthropometric data were measured according to standard methods. The body mass index (BMI) and waist to hip ratio (WHR) were calculated according to the standard protocol of the World Health Organization (WHO), based on minimal clothing and no shoes, using Falcon scales (Seca, Hamburg, Germany), measured to the nearest 0.1 kg for weight, and the nearest 0.1 cm for length/height. Hip circumference (HC) was measured at the widest part of the buttocks, and waist circumference (WC) was measured at the midpoint between the last rib and the iliac crest (umbilical level). BMI and WHR were calculated based on the following formula: weight (kg)/height (m^2^), and WC (cm)/HC (cm), respectively [17].

### Dietary assessment

Dietary intake was assessed by using a semi-quantitative Food Frequency Questionnaire (FFQ). The validity of this questionnaire has previously been confirmed in Iranian populations [18]. The FFQ included 168 food items, which was designed according to the frequency of consumption of the common foods of one’s country during the preceding 12 months (number of times consumed daily, weekly, monthly, and annually). The FFQ was filled out by a trained dietitian, through remote interviewing. The total meat consumption was defined as red meat, poultry, fresh fish, canned fish, processed meats, and organ meats. Information on alcohol use was not collected for cultural reasons and was therefore not analyzed. The dietary habits of each person were assessed one year prior to infertility diagnosis.

### Semen analysis

Semen samples were collected following 3 days of abstinence. Before transferring the samples into the container, the temperature of the container was matched to the body temperature of 37 °C. Semen samples were kept in sterile containers at 37 °C for 30 min, and were then evaluated and analyzed according to the WHO fifth edition laboratory guidelines [19]. Four parameters related to semen and sperm, including semen volume, sperm concentration, normal sperm morphology, and sperm motility were measured.

### Statistical methods

In the present study, the consumption of different types of meat was divided into four categories (quartiles). Then, the relationship between these four categories with the variables of age, body mass index, education, smoking, physical activity, and the social-economic index was measured by Chi-square and One-way Analysis of Variance (ANOVA) tests. Next, the relationship between meat consumption and semen quality indicators of participants was investigated by using Linear mixed models, adjusted for age, BMI, WHR, physical activity, smoking, and SES status, using pesticides, as well as macro and micro-nutrients intake. A *P*-Value of less than 0.05 was considered to represent statistical significance. All statistical analyses were performed using SPSS software (version 22, Chicago, IL, USA).

## Results

Table 1 details the main demographic information of men included in the analysis, according to quartiles of meat consumption. In accordance with Table 1, there were significant differences among quartiles of total animal flesh food consumption in terms of age, BMI, WHR, SES, smoking status, physical activity, and nutrients intake. Further, total animal flesh food consumption was positively related to total energy intake.

**Table 1:** Characteristics of 400 infertile men (number (%) unless stated otherwise)

Variables		Total meat intake				
		Quartile 1	Quartile 2	Quartile 3	Quartile 4	***P***-value^a^
Range (Serving/day)		0–0.96	0.97–1.32	1.32–2.15	2.16–4.57	
Number of participants		109	91	101	99	
**Participants demographic information**						
Age (year)		32.44(31.84–33.00)	35.31(34.57–36.05)	35.33(33.93–36.03)	31.80(31.34–32.26)	< 0.001
BMI kg/m^2^		23.74(23.36–24.12)	28.24(27.82–28.66)	27.47(26.76–28.18)	25.44(24.99–25.89)	< 0.001
Waist to Hip Ratio		0.94(0.91–0.97)	1.13(1.09–1.17)	1.08(1.03–1.13)	0.96(0.93–0.99)	< 0.001
Socioeconomic		4.81(4.62–5)	4.99(4.84–5.14)	4.40(4.22–4.58)	3.85(3.61–3.91)	< 0.001
Education (years)		9.10(8.57–9.63)	10.22(9.88–10.56)	12.21(11.79–12.63)	3.85(3.61–4.09)	< 0.001
Number of participants used pesticide		79(66%)	67(73%)	51(50%)	59(59%)	< 0.001
Smoking	Never	49 (44%)	55 (60%)	62 (61%)	52 (52%)	0.006
	Past smoker	1 (0.09%)	3 (3%)	5 (4%)	9 (9%)	
	Current smoker	59 (54%)	33 (36%)	34 (33%)	38 (38%)	
Physical activity	Low	46(42%)	25(27%)	45(44%)	20(20%)	< 0.001
	Moderate	35(32%)	13(14%)	88(87%)	21(21%)	
	Extreme	54(49%)	0(0%)	21(20%)	32(32%)	
Diet	Total energy (kcal/day)	1555(1300–1810)	1960 (1630–2290)	2330 (2020–2650)	2826 (2566–3086)	< 0.001
	Protein (% of energy)	16.5 (13.1–19.9)	16.3 (12.7–19.9)	16.6(13.1–20.1)	16.1 (13.1–19.1)	< 0.001
	Fat (% of energy)	24 (20.3–26.7)	31 (24.1–37.9)	33 (30.5–35.5)	38 (35.8–40.2)	< 0.001

^a^From the ANOVA test for continuous variables, Chi-square test for categorical variables

Consumption of canned fish was inversely related to sperm immotility. Compared with the men in the lowest quartile of canned fish intake, those in the highest quartile had 5.1% fewer sperm immotility [lowest quartile: 52.5%; (95% CI: 47–57) vs 47.4%; (95% CI: 43–51) P-trend = 0.026]. Similarly, a trend toward an inverse significant association between fresh fish intake and sperm immotility was observed ([lowest quartile: 51.9%; (95% CI: 48–55) vs 49.8%; (95% CI: 45–54) P-trend = 0.0.074]; however, this relationship was not linear among quartiles. In contrast, fresh and canned fish intake was unrelated to other outcomes of sperm quality (P-trend > 0.05). Furthermore, we failed to detect any association between consumption of processed total meat, red meat, red meat, poultry, and organ meat, and semen quality indicators (P-trend > 0.05) (Table 2).

**Table 2:** The relation between semen quality indicators in 400 infertile men and intake of different meat types

Meat intake (servings/day)	Semen volume (ML)	Total sperm count (million)	Progressive motility (% motile)	Sperm morphology (% normal)	Sperm immotile (% immotile)
**Total meat**					
Quartile 1	3.35 (3.0–3.7)	37.5 (30–44)	28.8 (26–31)	2.4 (2.2–2.7)	49.3 (45–53)
Quartile 2	3.4 (3.1–3.7)	40.6 (34–47)	32.5 (29–35)	2.6 (2.4–2.9)	51.8 (48–55)
Quartile 3	3.7 (3.3–4.0)	41.3 (35–48)	30.9 (28–33)	2.5 (2.2–2.7)	50.1 (46–53)
Quartile 4	3.8 (3.4–4.1)	43.8 (37–50)	31.7 (29–34)	2.6 (2.4–2.9)	52.8 (49–56)
*P*-value	0.173	0.615	0.302	0.636	0.528
**Processed red meat**^**a**^					
Quartile 1	3.7 (3.3–4.1)	43.3 (36–50)	31.2 (28–34)	2.5 (2.2–2.7)	48.3 (44–52)
Quartile 2	3.2 (2.7–3.6)	37.7 (29–44)	28.7 (25–31)	2.5 (2.2–2.8)	49.5 (45–53)
Quartile 3	3.7 (3.3–4.1)	41.4 (34–49)	31.4 (28–34)	2.5 (2.3–2.8)	54.9 (50–58)
Quartile 4	3.6 (3.2–4.1)	39.8 (32–46)	32.1 (29–35)	2.6 (2.3–2.9)	51.8 (47–55)
*P*-value	0.195	0.623	0.282	0.856	0.132
**Red meat**^**b**^					
Quartile 1	3.6 (3.3–4.1)	41.6 (34–47)	30.2 (27–33)	2.5 (2.3–2.8)	49.4 (45–53)
Quartile 2	3.5 (3.1–3.8)	42.3 (35–48)	30.4 (27–33)	2.6 (2.3–2.9)	53.6 (49–56)
Quartile 3	3.2 (2.9–3.6)	37.4 (30–44)	30.7 (27–33)	2.5 (2.2–2.8)	51.4 (48–55)
Quartile 4	3.7 (3.4–4.1)	42.5 (36–49)	32.4 (29–35)	2.5 (2.2–2.8)	49.8 (45–53)
*P*-value	0.202	0.697	0.729	0.958	0.476
**Poultry**^**c**^					
Quartile 1	3.9 (3.4–4.3)	41.5 (33–50)	30.8 (27–34)	2.5 (2.1–2.8)	50.1 (45–54)
Quartile 2	3.2 (2.8–3.6)	35.1 (27–42)	29.8 (26–33)	2.5 (2.2–2.9)	49.5 (45–53)
Quartile 3	3.4 (3.1–3.8)	41.8 (35–47)	31.1 (28–33)	2.5 (2.3–2.8)	51.1 (47–54)
Quartile 4	3.7 (3.2–4.2)	45.6 (35–55)	32.8 (28–37)	2.6 (2.2–3.1)	53.7 (48–59)
P-value	0.197	0.353	0.741	0.944	0.655
**Fresh fish**					
Quartile 1	3.5 (3.1–3.9)	39.2 (31–46)	31.1 (27–34)	2.3 (2.0–2.6)	51.9 (48–55)
Quartile 2	3.5 (3.1–3.8)	42.4 (35–48)	30.9 (28–33)	2.5 (2.2–2.8)	45.3 (39–50)
Quartile 3	3.5 (3.2–3.9)	40.1 (33–47)	30.6 (27–33)	2.7 (2.4–3.0)	53.8 (50–57)
Quartile 4	3.6 (3.2–4.1)	41.7 (33–49)	31.7 (28–35)	2.7 (2.4–3.0)	49.8 (45–54)
*P*-value	0.955	0.930	0.969	0.341	0.074
**Organ meat**^**d**^					
Quartile 1	3.8 (3.4–4.1)	42.7 (36–49)	32.1 (29–34)	2.6 (2.3–2.9)	51.7 (47–56)
Quartile 2	3.3 (2.8–3.8)	35.5 (26–45)	27.7 (23–31)	2.3 (1.9–2.7)	49.3 (45–53)
Quartile 3	3.3 (3.0–3.7)	39.5 (32–46)	31.1 (28–33)	2.6 (2.3–2.8)	50.7 (46–54)
Quartile 4	3.5 (3.1–3.9)	42.8 (35–50)	31.4 (28–34)	2.6 (2.2–2.9)	52.5 (48–56)
P-value	0.251	0.603	0.371	0.648	0.713
**Canned fish**					
Quartile 1	3.4 (2.9–3.9)	41.6 (32–51)	33.6 (29–37)	2.7 (2.3–3.1)	52.5 (47–57)
Quartile 2	3.4 (2.9–3.9)	35.7 (26–45)	28.3 (24–32)	2.2 (1.8–2.6)	53.1 (49–46)
Quartile 3	3.6 (3.3–4.0)	38.6 (31–46)	29.1 (25–32)	2.5 (2.1–2.8)	49.7 (44–54)
Quartile 4	3.5 (3.2–3.8)	43.7 (37–49)	32.3 (29–34)	2.7 (2.4–2.9)	47.4 (43–51)
*P*-value	0.824	0.587	0.120	0.247	0.026

^a^Processed red meat included sausages and bologna

^b^Red meat included hamburger, beef and lamb meat as a mixed or main dish

^c^Poultry included chicken with or without skin, as a main dish, sandwich, or frozen dinner

^d^Organ meat included liver and chicken liver

## Discussion

The present cross-sectional study sought to investigate the relationship between animal flesh food consumption and semen quality indicators. The results of this study indicate an inverse association between canned fish consumption and sperm immotility. In addition, a trend toward an inverse significant association between fresh fish intake and sperm immotility was also observed. However, the other variables in meat groups, including intake of total meat, processed red meat, red meat, poultry, and organ meat did not show any significant association with any semen quality indicators.

Male infertility is an important disorder that can deleteriously impact both productivity and quality of life. Several studies have reported on the relationship between meat consumption and semen quality parameters as a proxy for male fertility [8, 14, 20, 21]; however, there are discrepancies among findings reported in the literature. Indeed, relatively small sample sizes in previous studies, in addition to the effect of ethnic differences, might be the cause of inconclusive findings, which highlights the necessity of further study to yield a reliable conclusion.

The current study highlighted an association between canned fish intake and sperm immotility. Studies that have evaluated the relationship between meat consumption and sperm quality indicators are limited and their findings are not consistent with the results of the present study [7, 9, 10, 14, 22]. A study conducted by Afeiche et al. [14] indicated that high consumption of processed meat was associated with a reduction of total sperm count and a progressive motile count. In addition, organ meat consumption, in particular, was related to higher total sperm count, higher sperm concentration, and greater sperm motility. Furthermore, additional empirical investigations have indicated that high consumption of organ meat may be associated with higher total sperm count, concentration, and motility [14, 20]. Indeed, it has been suggested that nutrients concentrated in organ meats, such as vitamin B12, iron, animal fat, animal protein manganese, and copper, conceivably explain these associations and may have a role in spermatogenesis [20]. A study in Boston, Unites States, found that higher fish consumption was associated with increased sperm count and normal sperm morphology [14]. Another study in the Netherlands found that fish and seafood consumption are effective in improving sperm motility and can increase the percentage of motile sperm [10]. One study in Iran reported that high consumption of processed meats is associated with an increased risk of developing asthenospermia, as compared to low consumption. However, among men who were ranked in the highest tertile of fish and seafood consumption, asthenospermia was less in comparison with those in the lowest tertile [7].

According to our findings, a greater consumption of canned fish was associated with a decrease in the percentage of immobile sperm. Canned foods are one of the most important dietary items as rich sources of micronutrients [23]. Canned fish, particularly tuna, is also widely consumed by the general public because it is rich in polyunsaturated fatty acids (PUFAs) [23, 24]. The polyunsaturated fatty acids in fresh fish are unstable and may oxidize rapidly [23, 25, 26]. One of the most prominent eating habits of residents of Yazd province, Iran, is the consumption of fried fish, in this cooking method, a large amount of polyunsaturated fatty acids is oxidized. Thus, the dietary intake of these fatty acids can be low, and may partly explain our results, which highlighted a trend towards an inverse significant association between fresh fish consumption and sperm motility. Therefore, dietary intake of fatty acids through canned fish may positively impact sperm motility due to the amount of available omega-3. Another possibility is that many pollutants, such as heavy metals, are highly stable, toxic, and not easily degradable in some of fish which are not selected for canning by manufacturers [27–29]. Indeed, continuous contact of the gastrointestinal tract with these toxins can affect the functioning of the human body system [23]. Among the various food groups, fish and its products contain higher levels of heavy metals than other groups, especially lead and cadmium, which can be harmful for sperm quality indicators [30].

Assessing patients’ follow-up and IVF/ISCI outcomes was not one of the goals of this study, and only studies on women have considered meat consumption and IVF/ISCI outcome [31]; therefore, more studies in this field are warranted to investigate the relationship between diet and IVF/ISCI outcomes, specifically in males.

This study has several strengths, including the recruitment of a large sample size of newly diagnosed infertile patients, with minimum error in dietary recall history, using an FFQ with high validity for estimating food intake and eating habits, and repeating semen analysis for reliability. However, the present study has some limitations that should be considered. The FFQ depends on the memory of the participants being interviewed, also it does not allow precise estimation of portion size of foods consumed. Moreover, the possible effect of stress as a confounder on men was not adjusted in this study. Despite the significant association found between meat consumption and infertility, which can be considered as a promising finding for nutritional policy related to this disorder, the observational nature study means that the findings cannot be generalized to a wider population nor does it permit causal inferences to be made. Indeed, the veracity of the results must be confirmed in further prospective studies with more diverse populations.

## Conclusion

In summary, the results from the present study suggest that canned fish consumption might be associated with improvements in sperm motility. Despite the novel findings of this study, further investigations are needed to confirm the veracity of these results and elucidate the mechanisms underlying the relation between meat consumption and male infertility.

## Supplementary information

**Additional file 1: Supplemental Figure 1.** Flow Chart of the patient recruitment.

## Data Availability

The datasets used and/or analyzed during the current study are available from the corresponding author on reasonable request.

## Abbreviations

IVF: In Vitro Fertilization
ICSI: Intracytoplasmic Sperm Injection
IPAQ: International Physical Activity Questionnaire
SES: Socioeconomic status
BMI: Body mass index
WHR: Waist to hip ratio
WHO: World Health Organization
HC: Hip circumference
WC: Waist circumference
FFQ: Food Frequency Questionnaire
ANOVA: One-way Analysis of Variance
PUFAs: Polyunsaturated fatty acids
