# Supplementary material for: The association between animal flesh foods consumption and semen parameters among infertile Iranian men: a cross-sectional study
Source: Nutr J. 2020 Oct 6;19:113. doi: 10.1186/s12937-020-00633-w (PMC7541170; doi:10.1186/s12937-020-00633-w)
Supplement: Supplementary file 1 — Additional file 1: Supplemental Figure 1. Flow Chart of the patient recruitment. [file 12937_2020_633_MOESM1_ESM.docx]

**Supplemental figure 1.** Flow Chart of the patient recruitment.

**Exclude:**

**Incomplete FFQ: 57**

**Inaccurate complement of FFQ: 34**

**Had chronic diseases: 17**

**Adhering to weight lost diet: 5**

**Final selected as participants**

**Exclude patients based on exclusion criteria**

**Initial recruitment of patients**
